# Long noncoding RNA profiling reveals that LncRNA BTN3A2 inhibits the host inflammatory response to *Eimeria tenella* infection in chickens

**DOI:** 10.3389/fimmu.2022.891001

**Published:** 2022-08-25

**Authors:** Hailiang Yu, Changhao Mi, Qi Wang, Guojun Dai, Tao Zhang, Genxi Zhang, Kaizhou Xie, Zhenhua Zhao

**Affiliations:** ^1^ College of Animal Science and Technology, Yangzhou University, Yangzhou, China; ^2^ Poultry Institute, Chinese Academy of Agricultural Sciences, Yangzhou, China

**Keywords:** chicken, *E. tenella*, lncRNA BTN3A2, inflammatory response, host resistance

## Abstract

Coccidiosis is a widespread parasitic disease that causes serious economic losses to the poultry industry every year. Long noncoding RNAs (lncRNAs) play important roles in transcriptional regulation and are involved in a variety of diseases and immune responses. However, the lncRNAs associated with *Eimeria tenella* (*E. tenella*) resistance have not been identified in chickens. In addition, the expression profiles and functions of lncRNAs during *E. tenella* infection remain unclear. In the present study, high-throughput sequencing was applied to identify lncRNAs in chicken cecal tissues from control (JC), resistant (JR), and susceptible (JS) groups on day 4.5 post-infection (pi), and functional tests were performed. A total of 564 lncRNAs were differentially expressed, including 263 lncRNAs between the JS and JC groups, 192 between the JR and JS groups, and 109 between the JR and JC groups. Functional analyses indicated that these differentially expressed lncRNAs were involved in pathways related to *E. tenella* infection, including the NF-kappa B signaling, B cell receptor signaling and natural killer cell-mediated cytotoxicity pathways. Moreover, through cis regulation network analysis of the differentially expressed lncRNAs, we found that a novel lncRNA termed lncRNA BTN3A2 was significantly increased in both cecum tissue and DF-1 cells after coccidia infection or sporozoite stimulation. Functional test data showed that the overexpression of lncRNA BTN3A2 reduced the production of inflammatory cytokines, including IL-6, IL-1β, TNF-α and IL-8, while lncRNA BTN3A2 knockdown promoted the production of these inflammatory cytokines. Taken together, this study identify the differentially expressed lncRNAs during *E. tenella* infection in chickens for the first time and provide the direct evidence that lncRNA BTN3A2 regulates the host immune response to coccidia infection.

## Introduction

The Avian coccidiosis is a widespread parasitic disease caused by *Eimeria* that causes huge economic losses to poultry production worldwide every year (1). After chickens ingest sporulated oocysts from the environment, the massive reproduction of coccidia destroys intestinal epithelial cells, resulting in chicken anemia, weight loss, bloody diarrhea and other symptoms, and even death in severe cases (2). Seven *Eimeria* species infect chickens, and *E. tenella* is one of the more pathogenic species. At present, due to the common use of anticoccidial drugs, large-scale death of chickens due to coccidial infection has rarely occurred, but the decline in chicken weight gain and egg production caused by recessive infection still brings serious economic losses to the poultry industry (3). Moreover, the use of anticoccidial drugs has led to the drug resistance and residues in chicken, and food safety issues (4). Vaccination can effectively prevent coccidiosis, but its application is limited due to high production costs.

Long noncoding RNAs are a class of RNAs longer than 200 nucleotides that rarely encode proteins (5). LncRNAs are widely involved in cell proliferation, apoptosis, various immune responses and pathological processes by interacting with mRNA, miRNA, DNA and proteins and other molecules (6–8). Recent studies have shown that lncRNAs play an important role in a host’s resistance to pathogenic infection (9, 10). For example, Riege et al. (11) showed that lncRNAs are involved in the regulation of vitamins A and D during bacterial and fungal infections. Dai et al. (12) revealed the lncRNA regulatory network during chicken *leukemia virus* infection. Moreover, lncRNAs can be involved in the infection of a host by a parasite (13). However, the expression and regulatory functions of lncRNAs during chicken *E. tenella* infection remain unclear.

In the present study, we identified lncRNAs in chicken cecal tissues from three groups (resistant, susceptible and normal groups) after *E. tenella* infection and investigate the functions of a key lncRNA (lncRNA BTN3A2, full-length sequence is 1397bp) to understand the role of lncRNAs and the complex regulatory mechanisms of host susceptibility and resistance to *E. tenella* infection.

## Materials and methods

### Animals and oocysts

Twenty healthy full-sib chicken families were randomly selected from Jiangsu Jinghai Poultry Industry Group Co., Ltd., and each family contained one male and four females. Each family was raised separately, and offspring were produced through artificial insemination (the number of F1 generations in each family was ≥ 10). The F1 generation chicks of each family were housed individually in sterile animal rooms and fed antibiotic-free feed and drinking water. At 18 days of age, the F1 individuals of each family were randomly divided into two groups (the challenge group and the control group). Each bird in the challenge group was orally challenged with 3.5×10^4^ *E. tenella* sporulated oocysts, and the control group was fed the same amount of PBS solution. According to the description in a previous study (14), on day 4.5 post-infection, the most resistant and most susceptible families were selected from 20 families by clinical symptoms (emaciation, drooping wings, and dying death), fecal scores and cecal lesion scores, respectively.

Coccidia oocysts were a gift from the Department of Parasites, College of Veterinary Medicine, Yangzhou University, and were expanded and rejuvenated in healthy 15-day-old chicks. The oocyst collection, purification and sporulation processes were described by Jiao et al. (15).

### Samples

On day 4.5 post-infection, 3 individuals from each group were randomly selected to be euthanized by cervical dislocation. The cecal tissues were collected and washed with sterilized PBS to remove the intestinal contents and mucus. Then, the treated cecal tissues were immediately stored in liquid nitrogen for future use. All the animal experimentation protocols were approved by the Animal Welfare Committee of Yangzhou University (license number: SYXK (Su) IACUC 2012-0029).

### Histopathology

The cecal tissues (JC, JS, and JR groups) were cut into approximately 0.5 cm pieces and fixed with 4% tissue fixative. After embedding in paraffin and sectioning (5 µm), the sections were stained with hematoxylin-eosin. Then, the slices were observed and imaged under a microscope (Nikon, Japan)

### Library construction and RNA sequencing analysis

Total RNA was extracted from cecum tissues with TRIzol reagent (Invitrogen, CA, USA) following the manufacturer’s recommended procedure. RNA purity and concentration were evaluated using a NanoDrop 2000 spectrophotometer (Thermo Fisher Scientific, Waltham, MA, USA), and RNA integrity was assessed using an Agilent 2100 Bioanalyzer (Agilent Technologies, Santa Clara, CA, USA). If the quality of the sample RNA met the experimental requirements (RNA integrity number>=7 and 28S/18S>=0.7), then subsequent sequencing was performed. Libraries were constructed with 1 µg of RNA from each sample using the TruSeq Stranded Total RNA with Ribo-Zero Gold Kit (Illumina, Cat. RS-122-2301) according to the manufacturer’s protocol. High-throughput sequencing, including lncRNA sequencing and mRNA sequencing, was carried out on an Illumina 2500 platform, and 150 bp paired-end reads were generated (OE Biotech, Shanghai, China).

### LncRNA identification and classification

The transcriptome from each dataset was assembled independently and then merged to generate a final transcriptome using the Cufflinks 2.0 program (16). All the transcripts that overlapped with known mRNAs, other noncoding RNAs and non-lncRNAs were removed. The obtained transcripts were screened according to a length greater than 200 bp and a number of exons >2. Next, CPC (v 0.9-r2) (17), PLEK (v 1.2) (18), CNCI (v 1.0) (19), and Pfam(v 30) (20) were used to predict and analyze the transcripts with coding potential. The novel predicted lncRNAs were obtained through these processes. The characteristics (including length, type, and number of exons) of the lncRNAs were analyzed after screening.

### Differential expression analysis of lncRNAs and mRNAs

Sequencing reads were mapped to the reference genome using HISAT2 (21). The novel predicted lncRNAs and known lncRNAs (from the NCBI and Ensembl databases) were subjected to differential expression analysis, and the expression of lncRNAs was calculated using fragments per kilobase of transcript per million mapped reads (FPKM) (22). The number of counts of each sample lncRNA was standardized using DESeq (23), and the negative binomial distribution test (NB) method was used to test the significance of the difference in the number of reads. For mRNA, HTseq-count (24) software was used to obtain the number of reads aligned to the protein-coding gene in each sample, and cufflinks was used to calculate the FPKM value of each gene. Finally, a fold change (FC) > 1 and a *P* value < 0.05 were set as the thresholds for significantly differential expression of lncRNAs and mRNAs.

### LncRNA functional analysis

To explore the functions of these differentially expressed lncRNAs during *E. tenella* infection, Gene Ontology (GO) enrichment analysis of the three categories of biological process (BP), cellular component (CP), and molecular function (MF) was performed on neighboring genes of the identified lncRNAs. Kyoto Encyclopedia of Genes and Genomes (KEGG) analyses were used to reveal enriched pathways of the differentially expressed neighboring genes. A *P* value ≤0.05 indicated significantly enriched GO terms and KEGG pathways.

### Coexpression network of the lncRNA-mRNA

The Pearson correlation coefficient (PCC) was used to analyze the correlation between differentially expressed lncRNAs and mRNAs and to construct a coexpression network. Coexpression relationship pairs of lncRNA-mRNA were selected according to a *PCC* value ≥0.85 and a *P* value <0.05, and a network map was then constructed using the R network package.

### Cis- and trans-regulation prediction of the differentially expressed lncRNAs

Studies have shown that lncRNAs regulate the expression of protein-coding genes through cis and trans methods (25). For cis-regulation prediction, the coding genes within 100 kb upstream and downstream of the differentially expressed lncRNAs and had significant co-expression with the lncRNAs were identified (*P ≤* 0.05). For trans-regulating prediction, the following procedures were used: (1) based on the coexpression results, we screened out lncRNAs and genes that were not on the same chromosome as the candidate targets, and (2) the number of bases for the two nucleic acid molecules of the lncRNA and gene to directly interact was ≥ 10, and the base binding free energy was ≤ -50. Then, the lncRNA cis- and trans-regulation networks were constructed using Cytoscape software.

### Cell culture and sporozoite exposure

DF-1 cells (a chicken embryo fibroblast cell line) were cultured at 37°C and 5% CO2 in basal medium (Gibco, USA) containing 10% fetal bovine serum (Gibco, USA) and 1% penicillin and streptomycin.

For sporozoite exposure, *E. tenella* sporozoites were separated from sporulated oocysts through the G3 funnel method. To ensure the successful construction of the *in vitro* cell coccidia model, the ratio of the number of sporozoites to chicken DF-1 cells was 3:1.

### LncRNA overexpression and interference vector

Three lncRNA BTN3A2-specific small interfering RNA (siRNA) and negative controls sequences (**Table 1**) were designed and synthesized by GenePharma Biotechnology (Shanghai, China). For overexpression, the lncRNA mature sequence (**Supplementary Text 1**) was inserted into the pcDNA3.1(+) cloning vector to construct the overexpression plasmid: pcDNA3.1-lncRNA BTN3A2.

**Table 1:** lncRNA BTN3A2 siRNA sequences and negative control sequence information.

SiRNA sequence	Sequences information (5’-3’)	Sequence size (bp)
siR-lncRNA BTN3A2-201	GCCAUGACAGGUGUUCCAATT UUGGAACACCUGUCAUGGCTT	21 21
siR-lncRNA BTN3A2-692	GCUAUUCCAGCCAUACUAATT UUAGUAUGGCUGGAAUAGCTT	21 21
siR-lncRNA BTN3A2-1000	GGGUCAUGAUGUUGUGUUUTT AAACACAACAUCAUGACCCTT	21 21
siR-NC	UUCUCCGAACGUGUCACGUTT ACGUGACACGUUCGGAGAATT	21 21

Chicken DF-1 cells were inoculated in a 12-well plate and grown to 70%. Then, the cells were subsequently transfected with 20 µmol/L siRNA or negative control, 0.8 µg/µl pcDNA3.1-lncRNA BTN3A2 and pcDNA3.1 using jetPRIME Transfection Reagent (Polyplus, Illkirch, France) for 24 h and then stimulated with sporozoites for another 6 h. The cells were harvested for RNA extraction, and the suspension was collected for ELISA.

### Real-time quantitative PCR (qRT-PCR)

Chicken cecal tissues from the JC, JR, and JS groups on day 4.5 post-infection were collected. Total RNA of tissue samples and DF-1 cells was isolated using TRIzol reagent for cDNA synthesis. Six differentially expressed lncRNAs in this study were selected for random verification of the RNA-Seq results, and β-actin was used as an internal reference gene. Primer sequences including chicken *IL-6*, *TNF-α*, *IL-1β* and *IL-8* (**Table 2**) were synthesized by Sangon Biotech Co., Ltd. (Shanghai, China). Then, qRT–PCR was performed using the ChamQ SYBR qPCR Master Mix Kit (Vazyme Biotech, Nanjing) according to the manufacturer’s specifications. The PCR program was run at 95°C for 30 s followed by 40 cycles of 95°C for 10 s and 60°C for 30 s. Each sample was analyzed in triplicate, and data analyses were performed using the 2−ΔΔCt method (26).

**Table 2:** The primer sequences for differentially expressed lncRNAs and Cytokines.

Name	Primer sequences (5’-3’)	Length (bp)
β-actin	F: CCTGAACCTCTCATTGCCA R: GAGAAATTGTGCGTGACATCA	152
TCONS_00025085	F: CGAGGGACAACGGGCAGAGCA R: GACGGAGTCGGAGGGGGAGCA	242
ENSGALT00000105766	F: CTGGAGAATGCTGACCCAGG R: AACCACCTACTAACGCTGGC	118
ENSGALT00000093710 (lncRNA BTN3A2)	F: CCCGACAGTTTCATGTGCAAG R: CTGCCAACTGGTGACATGGT	136
ENSGALT00000096203	F: GAGGGCAGAGGGCAGTATTT R: CTGTATCCCAAAGGGGCAGT	162
TCONS_00010899	F: TTTGCTCGACAAATCTGACCG R: CTGCAAACCAGCAATCCACA	250
TCONS_00007570	F: AAGAAGAAGAAGCGGCGTGG R: GCAAAAGGCCAACTACAGCAA	227
IL-6	F: AAATCCCTCCTCGCCAATCT R: CCCTCACGGTCTTCTCCATAAA	106
IL-1β	F: GGTCAACATCGCCACCTACA R: CATACGAGATGGAAACCAGCAA	86
TNF-α	F: GCCCTTCCTGTAACCAGATG R: ACACGACAGCCAAGTCAACG	74
IL-8	F: AGTTCATCCACCCTAAATCC R: CACACCTCTCTTCCATCCTT	106

bp, base pair; lncRNAs, Long Nocoding RNA; F, forward; R, reverse.

### Enzyme-linked immunosorbent assay

Chicken IL-6, TNF-α, IL-1β and IL-8 in the supernatant of DF-1 cells were measured by enzyme-linked immunosorbent assay (ELISA) according to the protocols of the kits (R&D Systems, USA).

### Statistical analysis

All the data are presented as the mean ± SEM, and one-way ANOVA *via* SPSS 25.0 software was used to compare the differences in data in the different groups. A *P* value < 0.05 was considered statistically significant.

## Results

### Chicken models of *E. tenella* infection

**Figure 1:**
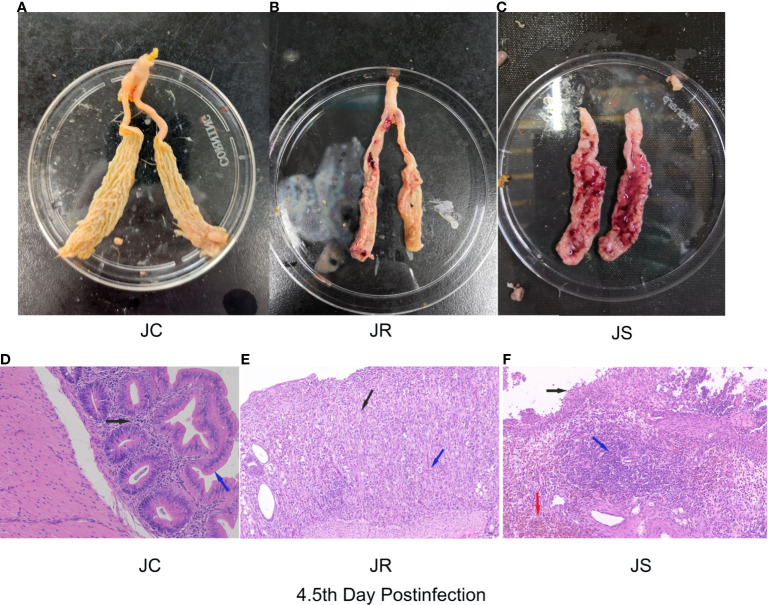
Histological and pathological images of cecal tissues of chickens at 4.5 days after *E. tenella* infection. **(A–C)** Images of chicken cecal tissues at 4.5 days post-infection in the JC group **(A)**, JR group **(B)**, and JS group **(C)**. **(D–F)** Pathological analysis of chicken cecal tissues at 4.5 days post-infection in the JC group **(D)**, JR group **(E)**, and JS group **(F)**.

Images of chicken cecal tissues at 4.5 days post-infection in the JC, JR and JS groups are shown in **Figure 1**. Bleeding spots appeared on the mucosal surface of the cecal tissue in the JR group. The cecum wall was slightly swollen and thickened, and the contents were relatively normal (**Figure 1B**). However, the chicken cecal tissues in the JS group had severe intestinal bleeding, and the intestinal wall was significantly swollen (**Figure 1C**). The cecal tissues of the control group are shown in **Figure 1A**. In addition, the pathological section analysis of the intestinal tissue in this study showed that the structure of each layer of the cecal tissue in the JC group was clear, the mucosal epithelium was intact, and the epithelial cells were tightly arranged (**Figure 1D**). The cecal tissue of the JR group chickens exhibited more mucosal necrosis (**Figure 1E**, black arrows) with moderate infiltration of lymphocytes and heterophils (**Figure 1E**, blue arrows). The cecal tissue in the JS group showed extensive mucosal necrosis and ulceration (**Figure 1F**, black arrows), a large number of lymphocytes (**Figure 1F**, blue arrows) and massive multifocal bleeding (**Figure 1F**, red arrows). Therefore, these data indicate that the *E. tenella* infection model in chickens of resistant and susceptible families was successfully constructed.

### Identification of lncRNAs in chicken cecal tissues by RNA-Seq

**Figure 2:**
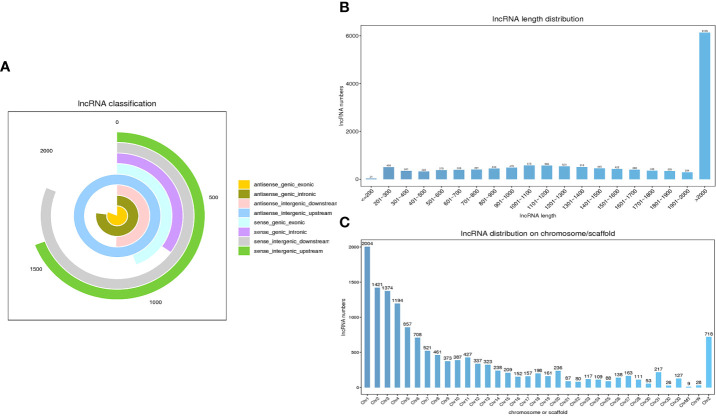
Characteristics of lncRNAs in chicken cecal tissues during *E*. *tenella* infection. **(A)** LncRNA classification. **(B)** The length distribution of the lncRNAs. **(C)** Chromosomal distribution of the lncRNAs.

After clean reads were compared to the chicken reference genome, a total of 13,809 lncRNAs were identified in the sequencing results from 9 samples. According to the source classification, the proportion of identified lncRNAs included exonic lncRNAs, intronic lncRNAs, intergenic lncRNAs and antisense lncRNAs as shown in **Figure 2A**. Moreover, the length distribution of the identified lncRNAs were shown in **Figure 2B**, and most of the lncRNAs were over 2000 bp in length. LncRNAs were distributed on every chromosome of chickens, and a large number of lncRNAs were distributed on chromosome 1, followed by chromosome 2 (**Figure 2C**).

### Differentially expressed lncRNAs in chicken cecal tissues infected by *E. tenella*

**Figure 3:**
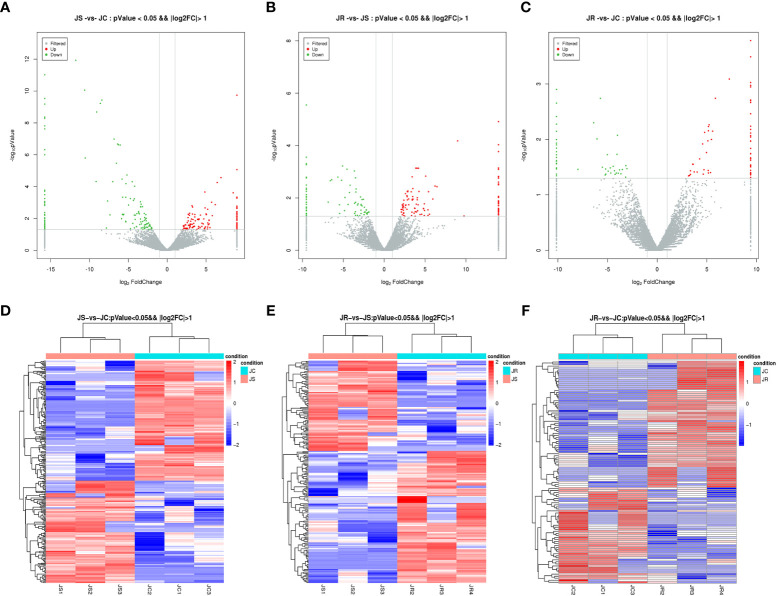
Expression profiles of differentially expressed circRNAs in chicken cecal tissues of different groups during *E*. *tenella* infection. **(A–C)** Volcano plots of differentially expressed lncRNAs in the JS vs JC group **(A)**, JR vs JS group **(B)**, and JR vs JC group **(C)**. **(D–F)** Hierarchical clustering plots of differentially expressed lncRNAs in the JS vs JC group **(D)**, JR vs JS group **(E)**, and JR vs JC group **(F)**.

To screen out the key lncRNAs during *E. tenella* infection, we analyzed the differential expression of the lncRNAs based on an FC > 1 and *P* value <0.05. A total of 192 lncRNAs were differentially expressed between the JS and JR groups, of which 114 were upregulated and 78 were downregulated; 263 differentially expressed lncRNAs were identified in the JS and JC groups, namely, 121 upregulated and 142 downregulated lncRNAs. Additionally, in the JR and JC groups, 109 differentially expressed lncRNAs were found, with 62 upregulated and 47 downregulated (**Table 3**; **Supplementary Table 1**; **Figures 3A–C**). Hierarchical clustering analysis revealed that the differentially expressed lncRNAs exhibited different expression patterns among the control group and the treatment groups (**Figures 3D–F**).

**Table 3:** Statistical information of differentially expressed lncRNAs.

Case	Control	Up numbers (DE lncRNAs)	Down numbers (DE lncRNAs)	Total DE lncRNAs (FC > 1, and the P-value < 0.05)
JC groups (JC1, JC2, JC3)	JS groups (JS1, JS2, JS3)	121	142	263
JS groups (JS1, JS2, JS3)	JR groups (JR2, JR3, JR4)	114	78	192
JR groups (JR2, JR3, JR4)	JC groups (JC1, JC2, JC3)	62	47	109

DE lncRNAs, differentially expressed lncRNAs.

### Functional analysis of the differentially expressed lncRNAs

**Figure 4:**
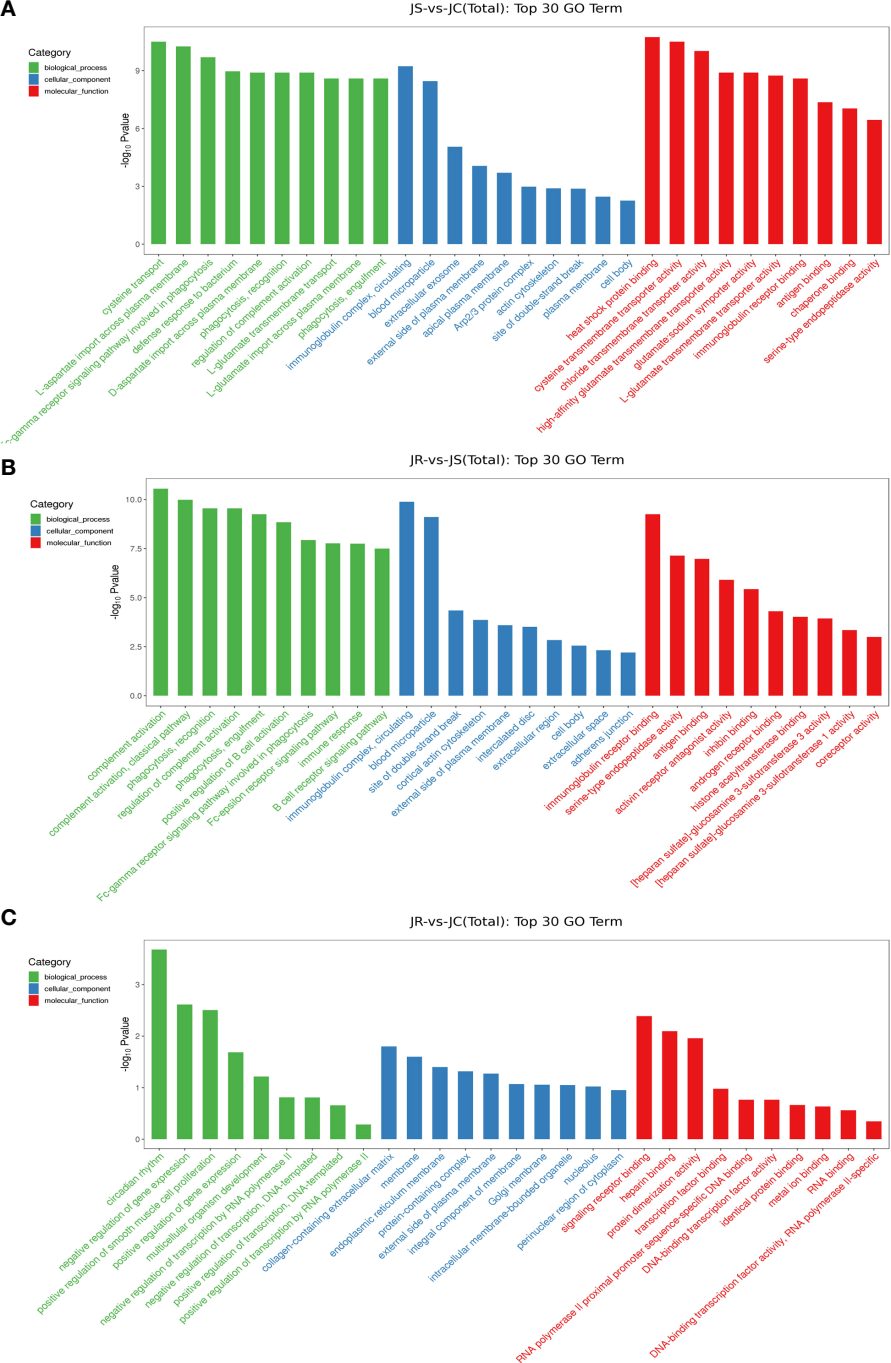
GO term analysis of the differentially expressed lncRNAs in chicken cecal tissues of different groups during E. tenella infection. **(A)** JS vs JC group. **(B)** JR vs JS group. **(C)** JR vs JC group.

To predict the potential functions of these differentially expressed lncRNAs, GO terms and KEGG pathways enrichment were analyzed according to the neighboring genes of the differentially expressed lncRNAs. The GO analysis results showed that the top 30 terms enriched in the JS vs JC groups mainly included bacterial defense response, circulating immune complexes and antigen binding. Similarly, the immune response, B cell receptor signaling pathway and immunoglobulin receptor binding were significantly enriched in the JS vs JR groups, while signaling receptor binding and transcription factor binding were significantly enriched in the JR vs JC groups (**Figure 4**; **Supplementary Table 2**).

**Figure 5:**
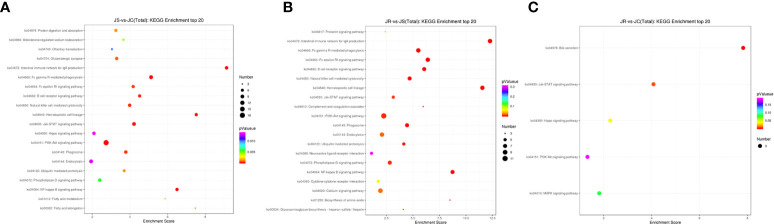
KEGG pathway analysis of the differentially expressed lncRNAs in chicken cecal tissues of different groups during E. tenella infection. **(A)** JS vs JC group. **(B)** JR vs JS group. **(C)** JR vs JC group.

The top 20 enriched KEGG pathways in the three groups that may participate in the coccidial immune response process mainly included the NF-kappa B signaling pathway, B cell receptor signaling pathway and natural killer cell mediated cytotoxicity in the JS vs JC and JR vs JS groups and the MAPK signaling pathway in the JR vs JC groups (**Figure 5**; **Supplementary Table 3**).

### Coexpression network analysis of the differentially expressed lncRNAs and mRNAs

**Figure 6:**
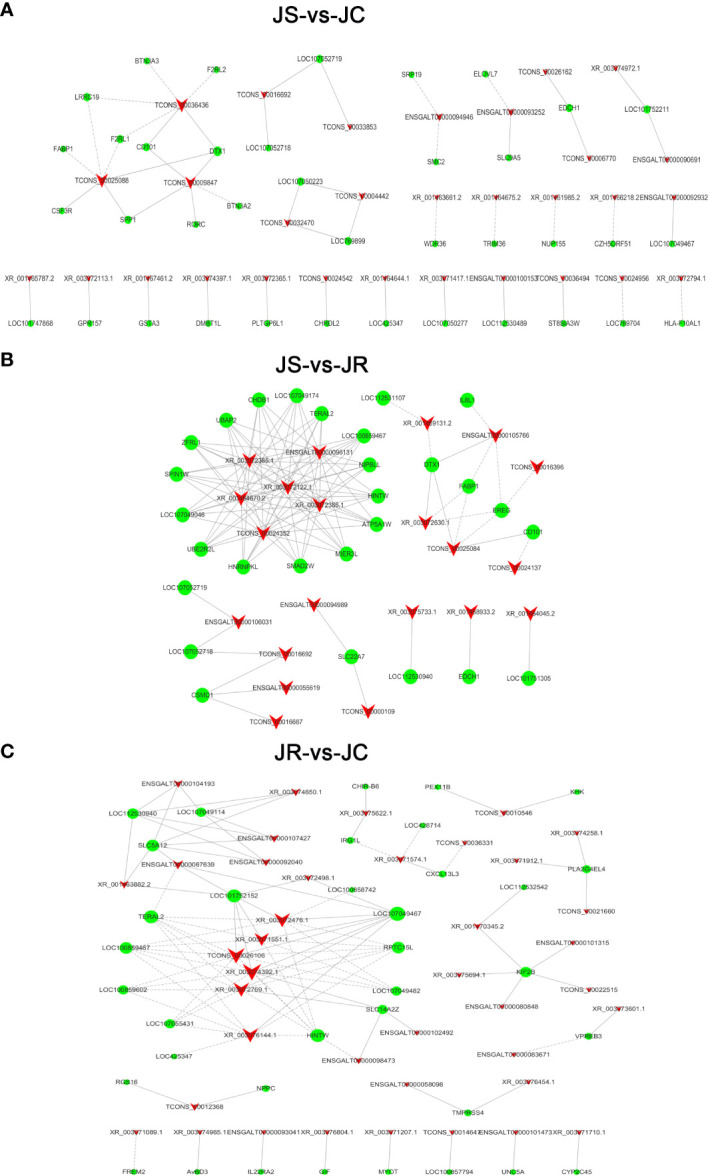
The coexpression network of differentially expressed lncRNAs and mRNAs. **(A)** JS vs JC groups. **(B)** JS vs JR groups. **(C)** JR vs JC groups. The solid lines indicate positive correlations, the dashed lines indicate negative correlations, the triangles represent the lncRNAs, and the circles represent the mRNAs.

To further reveal the function of lncRNAs during *E. tenella* infection, coexpression networks of the differentially expressed lncRNAs and mRNAs in the JS, JR and JC groups were constructed (**Figure 6**; **Supplementary Table 4**). In the JS vs JC groups, TCONS_00025088 had a significant negative coexpression relationship with the immune-related genes *LRRC19* and *F2RL1*. TCONS_00009847 was positively coexpressed with *SPP1* and *RORC*, and TCONS_00036436 was negatively coexpressed with *F2RL2* and *BTN3A3*. Moreover, XR_003072630.1 and TCONS_00025084 were coexpressed with *DTX1* and *FABP1* in the JR vs JS groups, and ENSGALT00000093041 was coexpressed with *IL22RA2* in the JR vs JC groups.

### Target gene prediction of the differentially expressed lncRNAs

**Figure 7:**
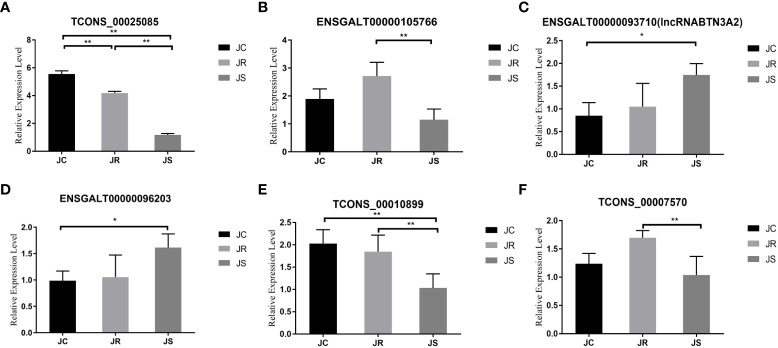
Validation of differentially expressed lncRNAs in chicken cecal tissues of different groups during *E*. *tenella* infection. The six lncRNAs were as follows: **(A)** TCONS_00025085, **(B)** ENSGALT00000105766, **(C)** ENSGALT00000093710 (lncRNA BTN3A2), **(D)** ENSGALT00000096203, **(E)** TCONS_00010899 and **(F)** TCONS_00007570. Values are the mean ± SD (n = 3 per group), and qRT–PCR analysis of three biological replicates was conducted. *P < 0.05, **P < 0.01.

For cis-targeted gene prediction, 20 mRNAs were cis-regulated by 24 lncRNAs in the JS vs JC groups, while 13 mRNAs were regulated by 12 lncRNAs in the JS vs JR groups, 5 mRNAs were regulated by 8 lncRNAs in the JR vs JC groups (**Supplementary Figure S1**). Interestingly, we found that BTN3A2, an important gene involved in immune and inflammatory responses, was a potential cis-target gene of ENSGALT00000093710 (termed lncRNA BTN3A2), and there was also a significant co-expression relationship (R=0.965, *P <*0.001). Moreover, this lncRNA was significantly increased in the cecum tissues after *E. tenella* infection (**Figure 7C**, *P <*0.05). Therefore, based on these evidences, we selected lncRNABTN3A2 for further functional experiments. In addition, the trans-targeted genes of the differentially expressed lncRNAs were also identified in **Supplementary Table 5**.

### Validation of differentially expressed lncRNAs

To verify the accuracy of the high-throughput sequencing results, we randomly selected six differentially expressed lncRNAs for qRT-PCR verification. The qRT-PCR results are consistent with the transcriptome sequencing results, which verifies the sequencing results of this study (**Figure 7**).

### Overexpression of lncRNA BTN3A2 inhibits host inflammatory responses after *E. tenella* infection

**Figure 8:**
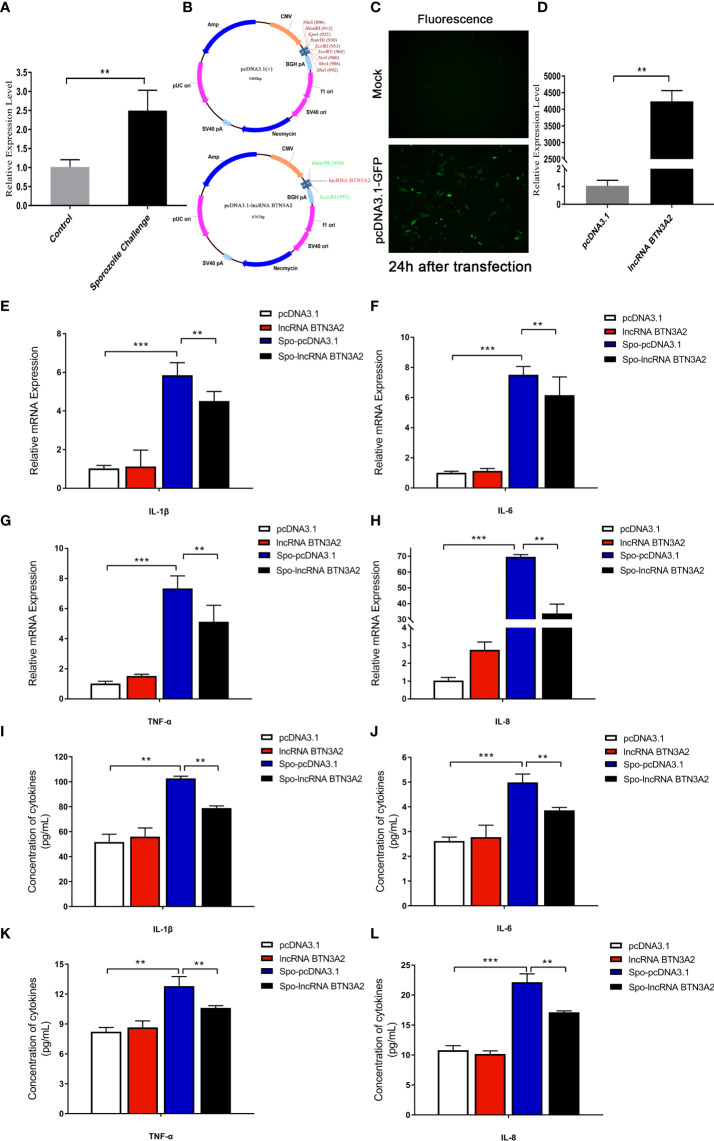
LncRNA BTN3A2 inhibits the production of inflammatory cytokines in sporozoite-stimulated DF-1 cells. **(A)** Expression of lncRNA BTN3A2 in blank and sporozoite-stimulated DF-1 cells. **(B–D)** Plasmid information **(B, C)** and overexpression effect **(D)** of lncRNA BTN3A2 after transfected DF-1 cells for 24 h **(E–H)** After 24 h of transfection, the cells were stimulated with sporozoite for another 6 h, and the mRNA expression levels of *IL-1β* **(E)**, *IL-6* **(F)**, *TNF-α* **(G)** and *IL-8* **(H)** were analyzed by qRT–PCR and normalized to β-actin. **(I–L)** After 24 h of transfection, the cells were stimulated with sporozoite for another 6 h, and the production of IL-1β **(I)**, IL-6 **(J)**, TNF-α **(K)** and IL-8 **(L)** in the supernatant of DF-1 cells was demonstrated by ELISA. *P < 0.05, **P < 0.01, and ***P < 0.001.

After coccidia infection or sporozoite stimulation, lncRNA BTN3A2 was significantly increased in chicken cecal tissue and DF-1 cells (**Figures 7C**, **8A**). This suggests that lncRNA BTN3A2 may be involved in the immune response to coccidial infection. Then, an *in vitro* model of *E. tenella* infection and the lncRNA BTN3A2 overexpression vector (pcDNA3.1-lncRNA BTN3A2) was constructed in this study (**Figure 8B**). The image of the fluorescent plasmid vector after transfection for 24 h showed that the pcDNA3.1 plasmid was successfully transferred into DF-1 cells (**Figure 8C**). As respect, the expression of lncRNA BTN3A2 in DF-1 cells was significantly increased when the cells were transfected with pcDNA3.1-lncRNA BTN3A2 compared to the NC group (**Figure 8D**). DF-1 cells were then stimulated with sporozoites for another 6 h, and the expression and protein levels of four inflammatory cytokines were detected. The results showed that sporozoite stimulation significantly increased the expression of *IL-1β*, *IL-6*, *TNF-α* and *IL-8* compared with the no-stimulation group. Moreover, the pcDNA3.1-lncRNA BTN3A2 further inhibited mRNA expression (**Figures 8E–H**) and the release (**Figures 8I–L**) of inflammatory cytokines, including IL-1β, IL-6, TNF-α and IL-8, in sporozoite-stimulated DF-1 cells.

### Knockdown of lncRNA BTN3A2 promotes the host inflammatory response to *E. tenella* infection

**Figure 9:**
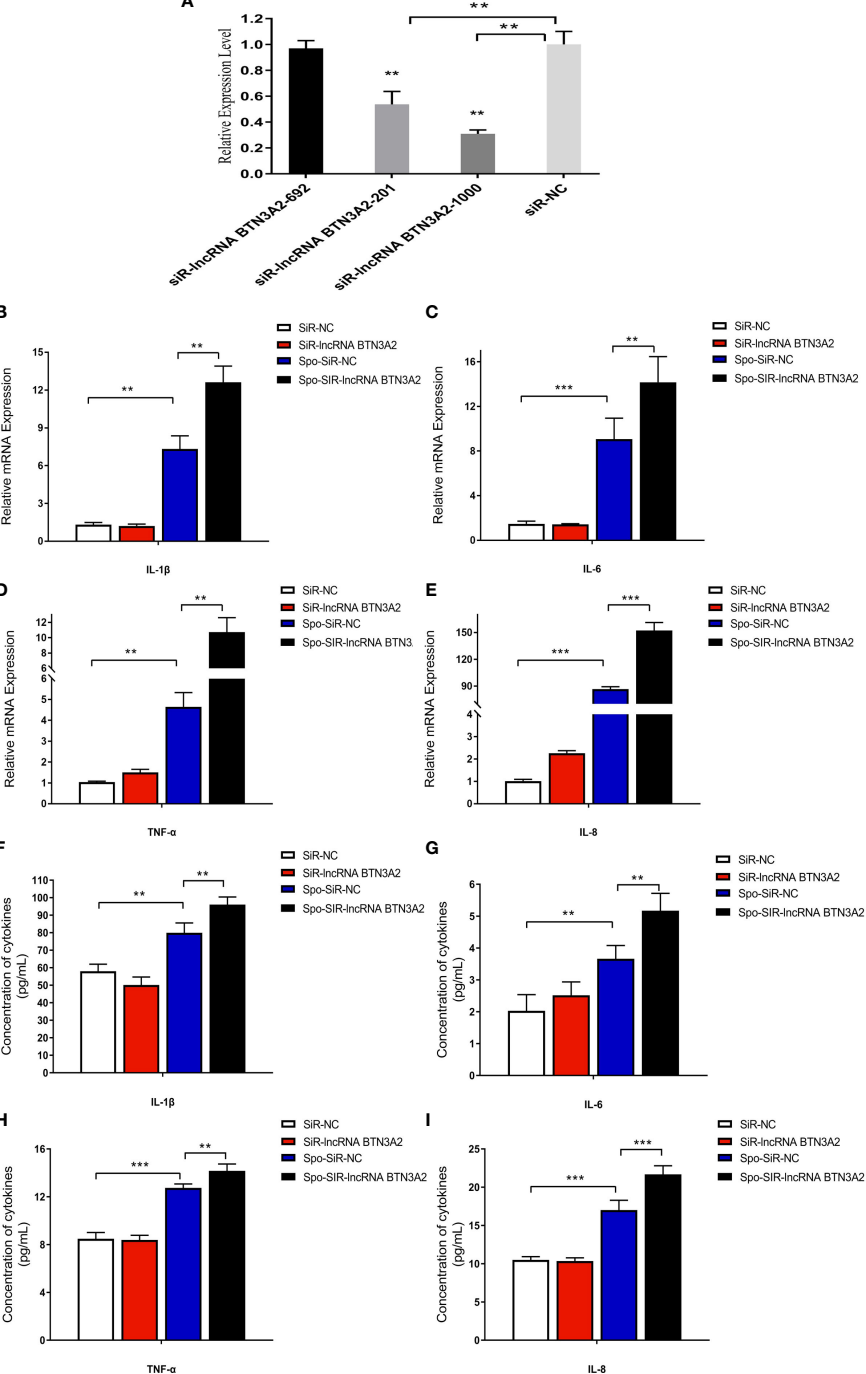
LncRNA BTN3A2 promotes the production of inflammatory cytokines in sporozoite-stimulated DF-1 cells. **(A)** Interference effect of three lncRNA BTN3A2 siRNA vectors after transfection with DF-1 for 24 h **(B–E)** After 24 h of transfection, the cells were stimulated with sporozoite for another 6 h, and the mRNA expression levels of *IL-1β* **(B)**, *IL-6* **(C)**, *TNF-α* **(D)** and *IL-8* **(E)** were analyzed by qRT–PCR and normalized to β-actin. **(F–I)** After 24 h of transfection, the cells were stimulated with sporozoite for another 6 h, and the production of IL-1β **(F)**, IL-6 **(G)**, TNF-α **(H)** and IL-8 **(I)** in the supernatant of DF-1 cells was demonstrated by ELISA. *P < 0.05, **P < 0.01, and ***P < 0.001.

To further confirm the effect of lncRNA BTN3A2 on the regulation of the inflammatory response, we silenced lncRNA BTN3A2 and then detected the release of inflammatory cytokines in sporozoite-stimulated DF-1 cells. After transfection with three interfering sequences of lncRNA BTN3A2 into DF-1 cells, siRNA-lncRNA BTN3A2-1000 had the highest interfering efficiency and significantly reduced the expression of lncRNA BTN3A2 (**Figure 9A**). DF-1 cells were then transfected with siRNA-lncRNA BTN3A2 to examine the production of inflammatory cytokines in sporozoite-stimulated DF-1 cells. The data showed that knocked down of lncRNA BTN3A2 further promoted the production of IL-6, IL-1β, TNF-α and IL-8 cytokines compared with siR-NC group (**Figures 9B–I**), which produced an opposite trend to that of lncRNA BTN3A2 overexpression. Taken together, these results further demonstrated that lncRNA BTN3A2 inhibits the host immune response against *E. tenella* infection, thereby avoiding excessive inflammation and immune responses.

## Discussion

Since the issue of policies to reduce the use of antibiotics in animal production, the impact of coccidiosis on the poultry industry has become more conspicuous. Therefore, many researches have focused on the immune response mechanism of the host infected by pathogens, to improve chicken disease resistance and production performance (27). The host gut defense consists of a series of barriers, including the gut microbiota barrier, the chemical barrier composed of the upper and lower mucus layers, the physical barrier of the intestinal epithelium, and the chemical barrier composed of a large number of immune cells (28–30). Obviously, the immune system is fundamental to keeping birds healthy. An effective gut immune system can rapidly respond to pathogenic attack to maintain tissue homeostasis and gut health (31). However, some chicken breeds and lines naturally exhibit differences in coccidiosis resistance and susceptibility that are influenced by genetic factors. These differences include the speed of immune-related cell responses and differences in immune gene expression (32–35). Therefore, understanding the immune differences between host resistance and susceptibility at the molecular level can be helpful for improving the host’s ability to resist pathogens.

Currently, studies have demonstrated that lncRNAs are involved in host immune responses, including innate immune and acquired immune responses. LncRNAs play an important role in the development of various diseases (cancer (36), cardiovascular disease (37), osteoporosis (38), atherosclerosis (39)) and inflammatory responses mediated by bacteria and viruses. We hypothesized that lncRNAs might be involved in the immune response during chicken *E. tenella* infection and play a role in host resistance and susceptibility. In this study, a total of 263 lncRNAs were differentially expressed in the JS vs JC groups, while 192 differentially expressed lncRNAs were identified in the JR vs JS groups and 109 in the JR vs JC groups. The results of this study showed that *E. tenella* infection induced significant alterations in the expression of these lncRNAs. Moreover, the functional pathways enriched by the adjacent genes of the differentially expressed lncRNAs were further investigated, and we found that the NF-Kappa B signaling pathway, B cell receptor signaling pathway and natural killer cell-mediated cytotoxicity pathway were significantly enriched in the JS vs JC and JR vs JS groups. The inflammatory response of the body after infection initiates the NF-Kappa B signaling pathway, which induces the expression of cytokines such as *IL-6*, *IL1β* and *TNF-a* to eliminate pathogen invasion (40, 41). The B cell receptor signaling pathway plays an important role in the process of antigen recognition, and NK cells are important immune cells that can kill target cells nonspecifically. These cells are involved in the body’s innate immune response after *E. tenella* infection (14, 42). Our studies suggest that these immune pathways may play a role in the defense of susceptible chickens against *E. tenella* infection. Furthermore, the MAPK signaling pathway was significantly enriched only in the JR vs JC groups. Studies have shown that the MAPK signaling pathway regulates biological processes such as cell growth, differentiation and apoptosis and is also closely related to diseases (43). The MAPK signaling pathway plays an important role in the interaction between Echinococcus multilocularis infection and host cells (44). The results of this study also revealed that the MAPK signaling pathway may be involved in immune regulation in resistant chickens.

The regulatory role of lncRNAs in inflammatory responses has always been a research hotspot. LncRNA Mirt2 is a negative regulator in the LPS-induced macrophage inflammatory response (45). LncRNA MIAT promotes inflammation and oxidative stress in sepsis-induced cardiac injury through the miR-330-5p/TRAF6/NF-κB axis (46). Moreover, lncRNA CDKN2B-AS1 acts as a molecular sponge of miR-195-5p and miR-16-5p to regulate the inflammatory response in ulcerative colitis (47). LncRNA-MAP3K4 is enriched in the vascular wall and regulates vascular inflammatory responses through the P38MAPK signaling pathway (48). In the present study, we found that lncRNA BTN3A2 was significantly increased after *E. tenella* infection compared to the control group. In addition, by analyzing the cis-targeted regulatory network map of the differentially expressed lncRNAs, lncRNA BTN3A2 targets the BTN3A2 gene and has a coexpression relationship. BTN3A2 is an important regulatory molecule in the immune response. BTN3A2 is required for the activation of gamma delta (γδ) T cells (49). BTN3A2 is also highly associated with CD8+ T cells, Th1 cells and dendritic cells and regulates immune infiltration of cancer through T-cell receptor interactions and nuclear factor signaling pathways (50). Therefore, lncRNA BTN3A2 may be involved in the immune response of chickens infected with *E. tenella*. Subsequently, functional test results showed that overexpression of lncRNA BTN3A2 significantly inhibited the production of inflammatory cytokines, including IL-6, IL-1β, TNF-α and IL-8, at the mRNA and protein levels. Conversely, knockdown of lncRNA BTN3A2 promoted the production of inflammatory cytokines. Therefore, our study showed that lncRNA BTN3A2 can inhibit the inflammatory response of a host infected with *E. tenella* and play an important role in the anticoccidial immune response of chickens. However, in this study, we do not show the effect and regulatory mechanism of lncRNABTN3A2 on target genes. As the cis-targeted regulatory mechanism of lncRNA has various ways, such as regulating the cis target genes through recruiting regulatory factors to the locus, repressing or activating cis chromatin, and through the DNA elements within the lncRNA promoter or gene locus (25), and these specific mechanisms will be revealed in the follow-up research of our group. In the long term, our findings provide novel references for anticoccidiosis measures, such as vaccine development and selection of anticoccidial breeds.

## Conclusion

In conclusion, this study firstly screened out the key lncRNAs and pathways, including the NF-kappa B signaling pathway, B cell receptor signaling pathway and natural killer cell-mediated cytotoxicity pathway during *E. tenella* infection. A novel lncRNA, lncRNA BTN3A2, was demonstrated to inhibit the production of inflammatory cytokines during coccidial infection. The research results were helpful to understand the complex mechanism of the chicken immune response to *E. tenella* infection and provide new insights into preventing and controlling chicken coccidiosis in the future.

## Data availability statement

The datasets presented in this study can be found in online repositories. The names of the repository/repositories and accession number(s) can be found below: https://www.ncbi.nlm.nih.gov/, PRJNA678759.

## Ethics statement

The animal study was reviewed and approved by Animal Welfare Committee of Yangzhou University (license number: SYXK (Su) IACUC 2012-0029).

## Author contributions

HY and GD designed the study and wrote the manuscript; HY, CM and QW performed the experiments, analyzed the data; HY and CM performed the transcriptome data and prepared the figures; TZ, GZ, KX, and ZZ reviewed the manuscript. All authors read and approved the final manuscript.

## Funding

This research was supported by the Yangzhou University International Academic Exchange Fund, the earmarked fund for Jiangsu Agricultural Industry Technology System. The Priority Academic Program Development of Jiangsu Higher Education Institutions (PAPD), and the earmarked fund for CARS-41.

## Acknowledgments

The authors would like to thank all members of this work for their advice and technical assistance.

## Conflict of interest

The authors declare that the research was conducted in the absence of any commercial or financial relationships that could be construed as a potential conflict of interest.

## Publisher’s note

All claims expressed in this article are solely those of the authors and do not necessarily represent those of their affiliated organizations, or those of the publisher, the editors and the reviewers. Any product that may be evaluated in this article, or claim that may be made by its manufacturer, is not guaranteed or endorsed by the publisher.
