# Supplementary figures and images for: Long noncoding RNA profiling reveals that LncRNA BTN3A2 inhibits the host inflammatory response to Eimeria tenella infection in chickens
Source: Front Immunol. 2022 Aug 25;13:891001. doi: 10.3389/fimmu.2022.891001 (PMC9452752; doi:10.3389/fimmu.2022.891001)

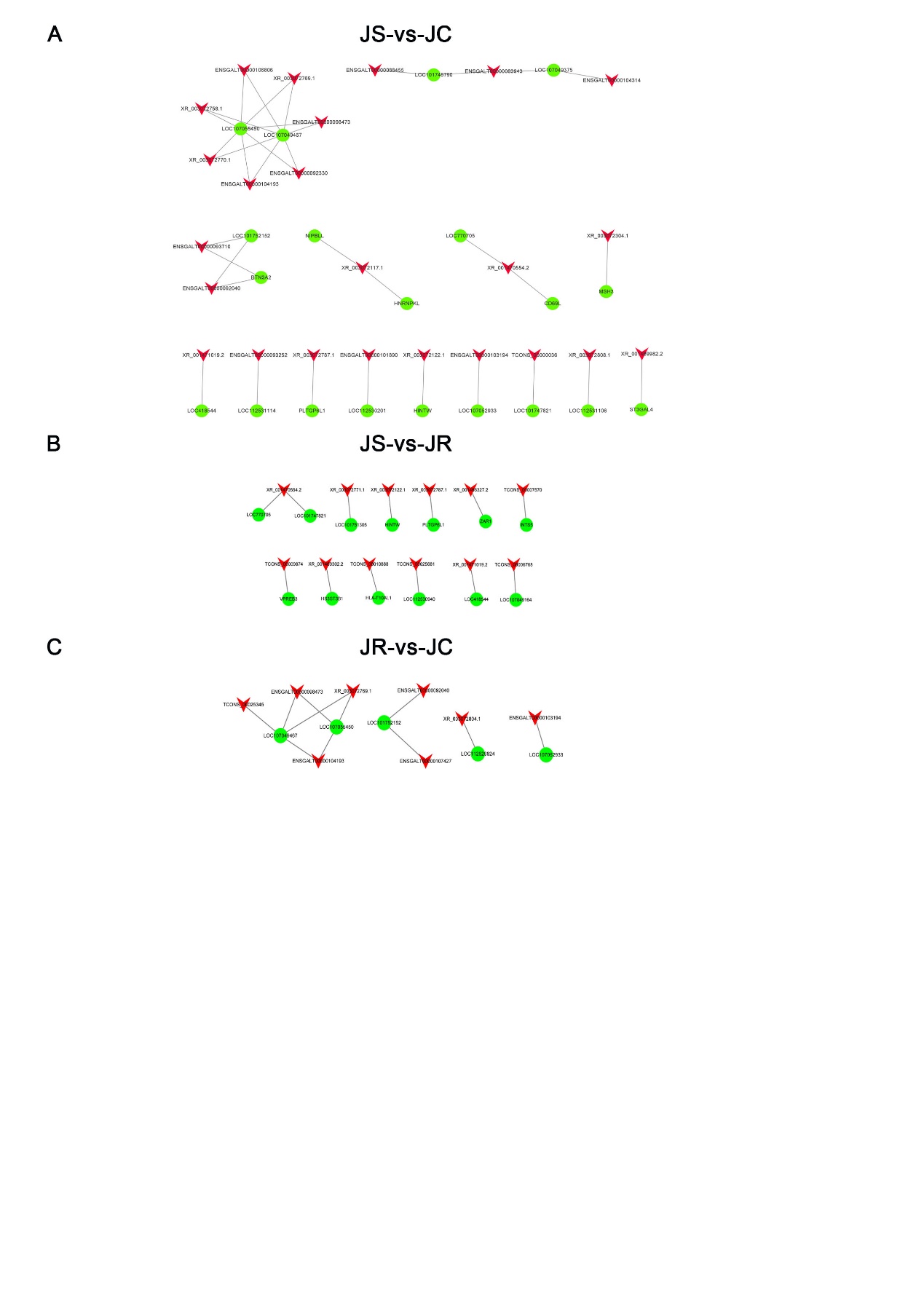
**Figure S1**

Supplement: Supplementary file 1 [file DataSheet_1.zip › Supplementary Figure S1.docx]
